# ‘We were treated like we are nobody’: a mixed-methods study of medical doctors’ internship experiences in Kenya and Uganda

**DOI:** 10.1136/bmjgh-2023-013398

**Published:** 2023-11-08

**Authors:** Yingxi Zhao, Daniel Mbuthia, David Gathara, Jacinta Nzinga, Raymond Tweheyo, Mike English, Kenya & Uganda Medical Internship Experience Study Group, Dos Santos Ankomisyani

**Affiliations:** 1 NDM Centre for Global Health Research, Nuffield Department of Medicine, University of Oxford, Oxford, UK; 2 KEMRI-Wellcome Trust Research Programme, Nairobi, Kenya; 3 MARCH Centre, London School of Hygiene and Tropical Medicine, London, UK; 4 Department of Health Policy Planning and Management, Makerere University School of Public Health, Kampala, Uganda; 5 Centre for Health Systems Research and Development (CHSRD), The University of Free State, Bloemfontein, South Africa; 1 Uganda Virus Research Institute - International AIDS Vaccine Initiative HIV Vaccine Program (UVRI-IAVI), Entebbe, Uganda; 2 NDM Centre for Global Health Research, Nuffield Department of Medicine, University of Oxford, Oxford, UK; 3 KEMRI-Wellcome Trust Research Programme, Nairobi, Kenya; 4 MARCH Centre, London School of Hygiene and Tropical Medicine, London, UK; 5 Kenya Medical Association, Nairobi, Kenya; 6 Kenya Medical Practitioners and Dentists Council, Nairobi, Kenya; 7 Kenyatta University School of Medicine, Nairobi, Kenya; 8 Nuffield Department of Primary Care Health Sciences, University of Oxford, Oxford, UK; 9 Department of Economics, Verona University, Verona, Italy; 10 Department of Surgery, Faculty of Medicine, Lira University, Lira, Uganda; 11 Department of Health Policy, Planning and Management, Makerere University School of Public Health, Kampala, Uganda; 12 Jomo Kenyatta University of Agriculture and Technology School of Medicine, Juja, Kenya; 13 Centre for Health Systems Research and Development (CHSRD), The University of Free State, Bloemfontein, South Africa; 14 University of Nairobi Faculty of Health Sciences, Nairobi, Kenya; 15 Department of Paediatrics and Child Health, University of Nairobi, Nairobi, Kenya; 16 Kenya Paediatric Research Consortium (KEPRECON), Nairobi, Kenya

**Keywords:** Health policies and all other topics, Health systems, Mental Health & Psychiatry

## Abstract

**Objective:**

Medical interns are an important workforce providing first-line healthcare services in hospitals. The internship year is important for doctors as they transition from theoretical learning with minimal hands-on work under supervision to clinical practice roles with considerable responsibility. However, this transition is considered stressful and commonly leads to burn-out due to challenging working conditions and an ongoing need for learning and assessment, which is worse in countries with resource constraints. In this study, we provide an overview of medical doctors’ internship experiences in Kenya and Uganda.

**Methods:**

Using a convergent mixed-methods approach, we collected data from a survey of 854 medical interns and junior doctors and semistructured interviews with 54 junior doctors and 14 consultants. Data collection and analysis were guided by major themes identified from a previous global scoping review (well-being, educational environment and working environment and condition), using descriptive analysis and thematic analysis respectively for quantitative and qualitative data.

**Findings:**

Most medical interns are satisfied with their job but many reported suffering from stress, depression and burn-out, and working unreasonable hours due to staff shortages. They are also being affected by the challenging working environment characterised by a lack of adequate resources and a poor safety climate. Although the survey data suggested that most interns were satisfied with the supervision received, interviews revealed nuances where many interns faced challenging scenarios, for example, poor supervision, insufficient support due to consultants not being available or being ‘treated like we are nobody’.

**Conclusion:**

We highlight challenges experienced by Kenyan and Ugandan medical interns spanning from burn-out, stress, challenging working environment, inadequate support and poor quality of supervision. We recommend that regulators, educators and hospital administrators should improve the resource availability and capacity of internship hospitals, prioritise individual doctors’ well-being and provide standardised supervision, support systems and conducive learning environments.

**WHAT IS ALREADY KNOWN ON THIS TOPIC**

- Ensuring appropriate and well-supported medical internship training is important for health workforce production and health systems’ quality of care, however, there is a scarcity of studies focusing on medical officer interns in low-ncome and middle-income countries where resources are most restrained thus education and working conditions are worst.

**WHAT THIS STUDY ADDS**

- Most interns are satisfied with their job but many reported working unreasonable hours as long as 72 hours due to staff shortage.
- Interns reported challenging scenarios where they had poor supervision and insufficient support due to consultants not being available, and sometimes interns were the only staff managing the wards or had to perform certain procedures unsupervised.
- Some consultants also expressed concerns with interns’ preparedness coming into the internship as well as competence postinternship.

**HOW THIS STUDY MIGHT AFFECT RESEARCH, PRACTICE OR POLICY**

- We highlighted the need to improve the resource availability and capacity of internship hospitals, ensure interns’ preparedness before internship, prioritise the well-being of individual doctors and ensure standardised supervision, support systems and conducive learning environments are in place.
- This study adds to the global literature on internship experiences of medical doctors and could also help others design evidence-based policies and interventions to address specific challenges during medical internships.

## Background

Medical interns are an important part of the workforce contributing to first-line healthcare service delivery in many hospitals. The internship year, often mandatory before full registration and licensing, is of great significance for medical doctors as they transition from theoretical learning and very highly supervised practical training to clinical practice roles with considerable responsibility.^1^ In many countries, this transition is associated with rapid development of burn-out and stress-related psychological problems due to challenging working conditions, continued learning and assessment.^2^ These and other factors influence interns’ competence, newly formed career identities,^3^ and the quality and safety of care being delivered.^4 5^ Additionally, today’s interns will become responsible for training the next cohorts of interns and other health worker cadres after they become fully qualified,^6^ thus poor internship experiences may be continuously passed on and compromise the quality of future training. Therefore, ensuring appropriate and well-supported internship training is important for health workforce production and health systems.

We previously conducted a scoping review on quantitative studies that measured internship experience and summarised three major and interconnected areas frequently examined by these studies,^7^ including (1) well-being which encompasses overall health and wellness, that is, the physical, mental and social health outcomes of interns including burn-out,^8–13^ stress,^14–17^ depression^14 16 18–20^ etc; (2) educational environment encompassing aspects which primarily influence where and how interns learn, that is, focused on the educational approach,^21–23^ supervision and mentorship^2 24 25^ and teamwork^26–30^ and (3) work conditions and environment aspects that primarily impact how interns work including terms and conditions of employment such as work hours, workload,^31^ safety,^32^ bullying and harassment^33^ and resources.^21^ Our review also highlighted the scarcity of studies focusing on interns in low-income and middle-income countries (LMICs) where resources, education and working conditions are poor.^7^ Specifically for Kenya and Uganda which have similar training and medical labour market contexts, we did not identify any published survey before 2020 that investigated internship training experience from the interns’ perspectives. More recently in May 2022, Makerere University in Uganda published its first Internship Situation Analysis Survey Report with responses from medical, dental and nursing interns, interviews with supervisors and patient representatives, and focus groups with interns.^34^ This highlighted that interns liked the internship period but faced specific challenges related to lack of equipment and materials, unavailable supervisors or inadequate support provided, as well as high levels of stress during the internship.^34^ This echoed one qualitative study in Kenya on medical graduates’ preparedness for internship responsibility as well as the working, training conditions and social support received by interns where deficient equipment and medication, inadequate accommodation and food provision were highlighted.^35^

In this paper, we present an overview of medical doctors’ internship experience in Kenya and Uganda using data from a questionnaire survey of 854 interns and junior medical officers (MOs) and semistructured interviews with 54 junior MOs and 14 consultants, collected between 2020 and 2021, using a mixed-methods approach to help understand internship experiences in major domains. This adds to the global literature on internship experiences of medical doctors and could also help researchers, medical educators, human resource managers and policymakers design evidence-based policies and interventions to address specific challenges during medical internship.

## Methods

### Study design and framework

Details of the methods are provided in table 1. In summary, we used a convergent mixed-methods approach^36^ to gain an in-depth understanding of the internship experience of junior MOs in Kenya and Uganda. We used quantitative survey to understand the extent of experiences with a larger population size, with the possibility of informing more routine survey methodology, and qualitative interviews to gain in-depth insights of potential differences in experiences. Data collection and analysis were guided by the three major themes identified in the scoping review,^7^ that is, well-being, educational environment and working conditions and environment. The mixed-methods approach included a quantitative questionnaire survey with current MO interns (who were still in their internship year) and junior MOs (who had completed their internship within 3 years) and qualitative semistructured interviews with junior MOs and their supervisors/consultants. Data collection was informed by the scoping review themes in both cases, but data were collected and analysed in parallel. Integration occurred at the data analysis and interpretation phase where we triangulated findings from both qualitative and quantitative data under each major theme.

**Table 1:** Data collection and analysis for quantitative survey and semistructured interview

	Quantitative survey data	Qualitative interview data
Instrument development	Questions identified by reviewing existing tools and indicators from the scoping review^7^ and five tools were included and adapted Postgraduate Hospital Educational Environmental Measure,^28^ Professional Quality of Life Scale,^58^ Perceived Stress Scale,^59^ Safety Attitude Questionnaire 60and Patient Health Questionnaire-9,^61^ with additional questions on physical resources. Context validity discussion with 18 medical interns in Kenya and Uganda, alongside 25 interns in 5 other LMICs, as well as through a 14-member expert panel validation. Pretesting and cognitive interviews with 19 medical interns across different countries including 6 in Kenya and Uganda.^38 39^ The final survey included 88 questions on well-being, educational environment and work condition and environment and 10 questions on sociodemographic and medical training. All internship experience-related questions were standardised into a five-point Likert scale, either ‘very often—often—sometimes—rarely—never’ or ‘strongly agree—agree—neutral—disagree—strongly disagree’.	Semistructured interview guide informed by the scoping review themes with additional questions on the broad context of internship such as COVID-19, health worker strikes and government decentralisation. For consultants we also asked them about their perception of interns’ preparedness and competence.
Data collection	We used a mix of convenience and snowball sampling approach for survey sampling. In Kenya, we engaged with a number of different stakeholders such as the Kenyan Medical Practitioners and Dentists Council, Kenya Medical Association Young Doctor Network, Kenya Young Doctor Caucus, three major medical schools and selected facilities in the Clinical Information Network operated by KEMRI-Wellcome Trust Research Programme, and asked them to share the survey through their respective platforms such as WhatsApp or SMS. In Uganda, the Federation for Uganda Medical Interns and the Uganda Medical Association were used for circulation of recruitment materials. The research team also physically visited five health facilities based in Kampala, and two major internship training sites upcountry and asked MO interns and junior MOs who work in these facilities to fill out the survey. At the time of data collection, one cohort of interns had just completed internship. Self-administered survey using REDCap tools either through participants’ smartphones or laptops in Kenya, or researcher-provided Tablets in Uganda. Data collection in Kenya started in Nov 2021 and ended in May 2022; in Uganda started in April 2022 and ended in June 2022.	Interviewees were sampled over June 2021 and June 2022 similarly using a mix of convenience and snowball approach, in Kenya, we identified several MOs and consultants through our study group network, and then asked them to further introduce us to more eligible participants in Kenya; In Uganda, recruitment was conducted by research assistants through health facility visits. We recruited 54 MOs who interned in different hospitals and currently working in different occupations, and 14 consultants from different specialties to understand the diverse experiences and perspectives. Interviews were conducted by research assistants who were researchers or MOs and had previous experiences of qualitative interviews. Interviews lasted 30–60 min and were conducted in English. They were mostly online in Kenya and face-to-face in Uganda. We felt that data saturation was achieved at the time of data collection.
Data analysis	A total of 845 respondents fully completed the questionnaire survey, out of 1222 starting the survey and 890 finished the survey (45 respondents have more than 10% missing). For quantitative data, we created binary categories by calculating the percentages of respondents that selected ‘strongly agree’ and ‘agree’ or ‘very often’ and ‘often’ for each item, and we presented the proportion for each item. We also compared responses to all items between different gender and graduation cohorts to understand potential differences.	We used a thematic analysis approach^42^ guided by the thematic framework identified from the scoping review. Codes were developed deductively and inductively based on the thematic frameworks, and within each thematic area, subthemes were further refined and summarised inductively. As the interview guide was relatively broad and there were new contextual subthemes emerging from the coding analysis that did not fit in the thematic framework, we listed these separately under ‘Health system internship training context and COVID-19’.
	For data integration, we organised the results by the three major themes and compared the results from survey questions with corresponding subthemes where relevant. This included using qualitative findings to validate or explain quantitative results.	

LMICs, low-income and middle-income countries; MO, medical officer.

### Data collection

For the quantitative questionnaire survey: a questionnaire was developed as part of a broader medical internship experience scale development and validation project with nine LMICs.^37^ Questions were identified using a deductive approach by reviewing existing tools and indicators from the scoping review.^7^ The questions from five existing tools previously used in other intern populations were further revised through content validity discussions, expert panel validation, pretesting and cognitive interview^38 39^ to ensure that all questions were relevant to internship training and that respondents could fully understand the questions. The survey questionnaire can be found in online supplemental appendix 1. We used a mix of convenience and snowball sampling approaches^40^ to include survey participants working with different stakeholders (see table 1 and online supplemental appendix 3 for more detail). For the semistructured interviews with junior MOs and consultants, we aimed to understand MOs’ internship training context and experiences. A semistructured interview guide (see online supplemental appendix 2) was developed based on the scoping review themes on well-being, educational environment and working conditions and environment. Recruitment and data collection strategies are different in Kenya and Uganda for both quantitative and qualitative components. Table 1 and online supplemental appendix 3 provide more detail on our recruitment and data collection strategies. It should be noted that our work was conducted during the COVID-19 pandemic, which presented both logistics challenges for data collection, but also may have affected internship training experiences which should be taken into consideration.

10.1136/bmjgh-2023-013398.supp1 Supplementary data

### Data analysis

For quantitative data, due to the ordinal nature and nonnormal distribution of Likert data, we present the percentages of respondents that selected ‘strongly agree’ and ‘agree’, or ‘very often’ and ‘often’ for each item. Some items are ‘positive items’ where agreement is interpreted as favourable thus the higher proportion the better experience; and some other items are ‘negative items’ where disagreement is interpreted as favourable thus the lower proportion the better experience.^41^ Descriptive analyses were performed using STATA V.17. Semistructured interviews were audiotaped and transcribed, with all personally identifiable information removed and replaced by unique anonymous codes, and imported into NVivo (V.1.4.1) for analysis. We used a thematic analysis approach to code the data,^42^ guided by the framework developed from the scoping review (see table 1). The integration process involved merging and comparing the results from the quantitative and qualitative data around the three thematic areas to provide a more complete understanding.^36^ Preliminary findings were presented to the other data collectors as well as all other authors who were not directly involved in coding to triangulate and increase the trustworthiness of the findings.

### Reflexivity

This study is a collaboration between Kenyan, Ugandan and international researchers. The first authors are a UK-based Chinese researcher (YZ) and a Kenyan researcher (DM) who are not doctors but with research experiences in public health, and the senior authors are a Ugandan doctor and researcher (RT) and a UK paediatrician with extensive experiences working in Kenya (ME). These four codesigned the study including instrument development and data interpretation to promote rigour. Data collection was conducted by DM and YZ in Kenya, and by RT and 10 other trained research assistants who are doctors or researchers in Uganda, transcripts were read by DM, YZ and RT, and data analysis was led by YZ with discussions with all other coauthors. Discussions were held between the first authors during the early stages of data collection to guide later interviews and analysis. Together with the 10 Ugandan short-term research assistants who are either doctors or researchers, and other Uganda-based and UK-based researchers, the research team included both ‘insiders’ and ‘outsiders’ thus were able to view study data from insider and outsider perspectives and engaged in conversations about what was going on.^43^ We also sense-checked these findings with external audiences including Kenya and UK-based health systems researchers. Online supplemental appendix 6 provides the structured reflexivity statement.

### Patient and public involvement

No patients were involved in this study.

## Results

A total of 845 respondents fully completed the questionnaire survey and 68 participated in the semistructured interviews. Sociodemographic characteristics of the survey participants can be found in table 2 and the interview participants in online supplemental appendix 4. The survey included respondents from different levels of internship hospitals. It should be noted that at the time of data collection, one cohort of Ugandan interns had just completed internship. The full results of the survey including means of each item stratified by gender, internship hospital level and graduation cohort are shown in online supplemental appendix 5.

**Table 2:** Survey sample characteristics

	Kenya (n=358)	Uganda (n=487)	Total (n=845)
Current status (%)			
Intern	36.6	13.4	23.2
Medical officer (completed internship)	63.4	86.6	76.8
Gender (%)			
Male	51.1	69.0	61.4
Female	47.5	30.8	37.9
Others/prefer not to say	1.4	0.2	0.7
Age (mean, SD)	28.4 (2.3)	28.0 (2.8)	28.2 (2.6)
Marital status (%)			
Single	80.7	83.6	82.4
Married	18.2	15.8	16.8
Divorced	0.3	0.2	0.2
Widowed	0.3%	0.4	0.4
Separated	0.6	0	0.2
Have children (%)	17.6	23.6	21.1
Months of internship training completed (for intern only, mean, SD)	6.5 (3.1)	2.5 (2.6)	5.2 (3.4)
Year of completing internship (for medical officer only, %)			
2022	22.9	48.3	39.5
2021	23.8	13.0	16.8
2020	26.4	21.1	23.0
2019	18.5	15.2	16.3
2018	8.4	2.4	4.5
Kenyan internship hospital level (%)			
Level four small (bed no <196)	27.7		
Level four large (bed no >196)	27.4		
Levels 5 and 6	38.3		
Others	6.7		
Uganda internship hospital level (%)			
General		24.0	
Regional referral		47.2	
National referral		25.1	
Others		3.7	
Funding for medical school (%)			
Completely self-funded	49.2	42.9	45.6
Received full/partial government scholarship	47.8	54.0	51.4
Others	3.1	3.1	3.1

### Well-being

As shown in table 3, most interns were satisfied with their internship job, evidenced by being able to help people (q17, 86% chose often or very often in Kenya and 85% in Uganda), believing they can make a difference through their work (q22, 77% and 86%) and proud of what they can do (q23, 82% and 87%). Most interns’ responses to stress, depression and burn-out-related questions were less positive. For example, 62% of Kenyan and 49% of Ugandan respondents often felt that they were unable to balance their work and personal life during the internship (q1) and 55% and 39% of Kenyan and Uganda interns, respectively, often felt worn out because of their internship work (q29). Kenyan interns scored most of their well-being items poorer than their Ugandan counterparts and within Kenya, interns in level 4 large hospitals had better scores (online supplemental appendix 5).

**Table 3:** Selected items and proportion of respondents that selected ‘agree’ and ‘strongly agree’ or ‘often’ and ‘very often’ from the questionnaire survey

Green items are ‘positive items’ where agreement is interpreted as favourable thus the higher proportion the better experience Red items are ‘negative items’ where disagreement is interpreted as favourable thus the lower proportion the better experience			Kenya (n=358)	Uganda (n=487)
Well-being	Q17	I get satisfaction from being able to help people.	86%	85%
	Q20	My ability to keep up with clinical techniques and protocols makes me feel pleased.	74%	84%
	Q22	I believe I can make a difference through my work.	77%	86%
	Q23	I am proud of what I can do to help as a medical intern.	82%	87%
	Q1	I feel that I am unable to balance my work and personal life during my internship.	62%	49%
	Q2	I feel nervous and/or stressed because of my internship work.	59%	38%
	Q8	I am angered because of things that were outside of my control.	50%	27%
	Q13	I feel tired or having little energy during my internship.	52%	27%
	Q29	I feel worn out because of my work as a medical intern.	55%	39%
	Q30	I feel overwhelmed because my case workload seems endless during the internship.	56%	42%
Educational environment	Q39	I have good clinical supervision at all times during my internship.	57%	66%
	Q46	I have enough clinical learning opportunities for my needs during the internship period.	62%	83%
	Q51	I have opportunities to acquire the appropriate practical procedures for clinical practice during my internship.	76%	81%
	Q52	My internship training makes me feel ready to be an independent medical practitioner.	77%	88%
	Q56	There is an informative and comprehensive internship guideline, log book or clinical diary.	82%	45%
	Q58	I feel part of a team working here.	71%	88%
	Q61	I have good collaboration with other medical practitioners, interns and clinical staff.	84%	88%
	Q54	I am pre-occupied with administrative work that impeded my ability to learn.	18%	17%
Work condition and environment	Q65	My work hours are appropriate during my internship.	19%	35%
	Q66	My workload is reasonable during my internship.	22%	32%
	Q71	There is a no-blame culture in my internship hospital.	23%	31%
	Q85	The internship hospital has good quality accommodation for me when on call.	35%	48%
	Q86	There are adequate catering services provided by the internship hospital when I am on call.	17%	34%
	Q87	The internship hospital has good internet connection for my study and work need.	23%	37%
	Q88	The internship hospital has adequate supply of diagnostics, equipment and medication for my study and work need.	34%	43%
	Q67	I am bleeped or called concerning the patients inappropriately during my internship.	51%	43%
	Q68	I have to perform inappropriate tasks during my internship.	39%	29%
	Q69	There is gender discrimination in my internship hospital.	16%	11%
	Q70	There are other forms of discrimination (eg, ethnicity, religion, tribe, disability) in my internship hospital.	27%	15%
	Q81	I get bullied or victimised within my internship hospital.	27%	13%

Data are presented as the proportion of respondents that chose ‘agree’ and ‘strongly agree’ or ‘often’ and ‘very often’. Items presented here are those of particular interest (have a high or low proportion, with difference between Kenya and Uganda or could be triangulated with qualitative data) and full results are shown in online supplemental appendix 5.

According to the qualitative interviews, emotional stress came from a variety of sources, including long working hours linked to the shortage of staff, first time facing deaths and breaking news to relatives, and for some moving into a new town. Additionally, Kenyan interns lived in constant fear of being ‘added weeks’ if they do not satisfy the requirement of supervisors. As internship requires supervisors to sign interns’ logbooks, they could ask interns to repeat some rotations and work additional weeks outside of the 12-month internship until the supervisors are satisfied. This was sometimes considered as being decided subjectively by the consultants. Furthermore, these added weeks are not paid so interns did not have any source of income if this sanction were imposed. Interns described ‘having burn-out is like the order of the day in internship’ (Kenyan medical officer, KMO07) or having burn-out is ‘automatic’ (Ugandan medical officer, UMO01), especially in some more intense rotations such as Obstetrics and Gynaecology. In comparison, while some consultants acknowledged that internship is indeed stressful, others commented that interns nowadays are not as resilient as the older generation and some considered the interns’ well-being and personal problems as ‘beyond my scope’ (Ugandan consultant, UC01).

‘I could say that…(burnout) got to a point where it was more of just pushing paper and not necessarily being keen to learn and gain the skills. Sometimes because you are tired you would find ways to make the work lighter for yourself which would mean sometimes making shortcuts just so that you are not too overburdened.’ (Kenyan medical officer, KMO04)

Some interns reported receiving emotional support from consultants, peers and friends, for example, through gathering after work or Bible study in one Kenyan Christian hospital. Other interns did not receive any support to relieve the pressure and were hesitant to discuss their well-being with their supervisors or direct colleagues. Interns suggested that more accessible mental health counselling during internship periods and coping strategy classes as part of the undergraduate curriculum would be helpful.

‘Basically there was no support, it is just you being, just adjusting to the pressure which is coming. You just adjusting and finding a way to loosen up or ease the pressure you are going through.’ (Kenyan medical officer, KMO05)

‘If you dared complain how tired you were, they would say you were lazy. The intern nurses would be switched three times before the intern doctors going home. Imagine you go to work at eight, at two, the nurse goes away and another comes up to five, at five you are on call and continue up to the following morning. if you say that you are tired, someone assures you how you are unemployable, lazy, bad attitude.’ (Ugandan medical officer, UMO06)

### Educational environment

Interns highly appreciated the clinical exposure during the internship. The survey results suggested that 76% of Kenyan interns and 81% of Ugandan interns agree that they have adequate opportunities to acquire the appropriate practical procedure skills for clinical practice (q51 as shown in table 3) and the internship training made them feel ready to become independent medical practitioners (q52, 77% and 88% agree or strongly agree in Kenya and Uganda, respectively). However, several interviewees in Uganda mentioned that where postgraduate training was provided in the same hospital, they were not able to practice certain skills as opportunities were given to postgraduate trainees, leaving them doing undesirable tasks.

‘The bad part about it was about the exposure because [Hospital A] has postgraduate training, especially in the surgical disciplines, you wouldn't get to do much because there are senior house officers year 1,2,3 …we used to fight physically to do any surgeries in theatres.’ (Ugandan medical officer, UMO15)

According to the questionnaire survey, over half of the interns felt that they had satisfactory clinical supervision at all times (q39, 57% and 66% in the Kenyan and Ugandan sample, respectively), however, supervision was rated lower in Kenya’s smaller hospitals (level 4 small) and Uganda’s larger hospitals (national referral) (see online supplemental appendix 5). Poor supervision due to consultants being unavailable was also reported by interviewees and sometimes interns were the only staff managing the wards or had to perform certain procedures unsupervised.

‘I’d hear some of my colleagues say they have already done several Caesarean sections, but via YouTube…they show up to a facility, they find they have no supervision and they’re the ones who are supposed to do the Caesarean sections.’ (Kenyan medical officer, KMO04)

In comparison, consultants in Kenya commented that they were already very stretched given the shortage of staff in the facilities thus they ‘cannot be with the intern at every point of the internship’ (Kenyan consultant, KC06) to supervise them. In response to interns’ comments of supervisors being too tough or giving them unreasonable demands, consultants indicated that current interns’ roles were much more relaxed than their own experience and interns’ expectation for constant supervision are ‘not realistic to what is on the ground’ (Kenyan consultant, KC07). Additionally, despite part of their job description, consultants commented that they do not receive any support or rewards from the hospital administration for providing supervision and some suggested that the Medical Council should provide some remuneration for supervisors.

In both countries, some consultants expressed concerns with interns’ preparedness coming into internship as well as competence postinternship. Some felt that interns are competent in making diagnoses and giving necessary treatment but lack a clear understanding of medical pathophysiology. However, some consultants perceived that 3 months internship rotations in each specialty were relatively short and that in some hospitals the huge number of interns challenged in-depth training, thus raising concerns regarding interns’ competence postinternship.

‘They go for a clinical, a child health rotation for about a couple of weeks, they do a new-born rotation for a couple of weeks, so in terms of practical… they have quite little time in the rotations. And that vis a vis the large numbers that enrolled for classes. You get individuals who are not able to take the practical aspect of their studies. So it’s sort of a conveyor belt. It’s just a large number that are being conveyed through the system.’ (Kenyan consultant, KC08)

As for teamwork and collaboration which are essential for a good educational environment, the survey results suggested that interns felt part of the team (q58, 71% of Kenyan respondents and 88% of Ugandan respondents agree or strongly agree) and had good collaboration with other doctors, interns and clinical staff (q61, 84% and 88%, respectively). This echoed some experiences of interns’ interaction with MOs according to the interviews. However, interview data also pointed out some occasions where collaboration with nurses and clinical officers was not always smooth. Senior nurses and specialised clinical officers in Kenya and senior house officers in Uganda sometimes looked down on interns and created a hostile environment for learning and working. Also due to staff shortages, interns sometimes felt they have to do nurses’ tasks as well.

‘…for me I always say it was the best because the team was friendly, they were ready to teach…as in when you walk in as an intern, you don’t have that fear that I am an intern, these guys are my seniors…so they were more approachable, more friendly and ready to teach.’ (Kenyan medical officer, KMO16)

‘What I didn’t enjoy was conflicts from some nurses who would object whatever decisions you arrived at, because they said that they are more experienced. They will do things a certain way so they go against whatever decisions you lay down.’ (Kenyan medical officer, KMO02)

Additionally, the role of internship coordinators was commonly discussed in the qualitative interviews with Kenyan interns. This specific role is usually carried out by a consultant in each facility that coordinates internship rotations, ensures there is a proper mechanism for supervision, positive educational environment and monitors the progress of the interns. Some interns reported that their internship coordinators were helpful and resolved their concerns, while others had poorer experiences where the coordinators were not being supportive. Such a role was not formally established in Uganda.

### Work conditions and environment

High workloads and long working hours were commonly reported by interns. According to the interview data, some interns were on call for 48 hours to 72 hours continuously or have to see a lot of patients every day in certain departments. Some interns felt that they were ‘treated as slaves to the hospital’ (Kenyan medical officer, KMO27). This is also in line with the quantitative findings where only 19% of Kenyan interns and 35% of Ugandan interns agreed that their work hours were appropriate (q65) and 22% of Kenyan interns and 32% of Ugandan interns agreed that their workload was reasonable (q66).

‘And then there was time there was only one intern in maternity, and they were expected to be on call every day for their entire rotation which is insane, and I think the guy even had to print a notice and just say ‘the intern is unavailable for work this week’ and just disappear because he said he was about to go mad.’ (Kenyan medical officer, KMO30)

‘However the obstetrics department is overwhelming and hectic. We worked too many hours for the obstetrics department… I would say one doctor is going to have to see like 200 mothers in one day.’ (Ugandan medical officer, UMO24).

In terms of bullying and discrimination, while only 16% Kenyan interns and 11% Ugandan interns agree with the existence of gender discrimination in their hospital (q69), other forms of discrimination were reported linked to ethnicity, religion, tribe or disability (q70, 27% and 15% agree or strongly agree) and some reported getting bullied or victimised within their hospital (q81, 27% and 13%). These reports were supported by interviewees suggesting that bullying and racial discrimination existed in some facilities.

‘I was racially profiled and at some point, it almost degenerated to physical abuse.…So I remember that morning it escalated into a very bad fiasco, this man flung a file at my face. And then you have nowhere to report it ‘cause they are people who are meant to sign you out and are people who are meant to give you a signature to leave. The same person who is your internship supervisor is actually deep kneading into the whole thing. (Kenyan medical officer, KMO08)

‘We were treated like we are nobody.… the big bosses, they treat you so bad. By treating you so bad I mean they humiliate you in front of patients or in front of any other people.’ (Ugandan medical officer, UMO19)

Over half of the interns felt accommodation, internet connection and diagnostics, equipment and medication supply were lacking. The lack of resources led to certain patients being referred to other hospitals thus interns not being able to acquire relevant skills, or interns forced to improvise.

‘We were highly understaffed, I remember when we hard to do a C- section wearing non surgical gloves. Gloves would go out of stock and then you had to improvise.’ (Ugandan medical officer, UMO13)

‘We have shortage of supplies. And that actually, you may find that you are preparing to do a procedure. And there are certain things that miss and because of the patients safety we just refer these patients to another hospital. So they miss out on the learning opportunities.’ (Kenyan consultant, KC02)

Interns also rated low on many patient safety-related questions. Due to staff shortage, many tasks that are supposed to be done by consultants were also conducted by interns including caesarean sections, admitting, discharging and reviewing patients. Nearly 39% of Kenyan and 29% of Ugandan interns thought that they had to perform inappropriate tasks during their internship (q68). Only 23% and 31% of interns in Kenya and Uganda thought that there is a no-blame culture in the facility (q71).

### Health system internship training context and COVID-19

Interns mentioned broad political and health systems contexts such as strikes, COVID-19 and decentralisation changes as impacting their internship experiences. Strikes led to spikes in workload in some internship hospitals and the closure of others. Additionally, COVID-19 posed varied scenarios, for some interns it led to fewer patients and elective procedures in hospitals, whereas some others managed more COVID-19 patients. Also in Kenya, interns commented that decentralisation led to upgrades of facility infrastructure but also in some counties delayed payment so that supervisors do not show up for work.

Noteworthily in Kenya, psychiatry and community health were added as two compulsory rotations for internship training by the Kenya Medical Practitioners and Dentists Council in 2020. However, interview data suggested that these rotations were rarely implemented in practice, reasons ranged from lack of a mechanism and structure for providing such rotations, to some internship coordinators’ intentional neglect. One intern commented that ‘there is nothing we did for the community, we just signed the log books.’ (Kenyan medical officer, KMO28)

‘Personally I don't, the government has not come up with proper structures about how we should do … There is the mental health that they introduced recently, the government has not come up with structures like okay you want us to do a mental health rotation for these students, do we take them to [Hospital AX]? Do we take them to [Hospital BN]? Do we take them to do counselling in the outpatient counselling department? There is no structure for, even in community health. Do we send them to a slum to go maybe, or do they during the polio vaccine, do they go to the community and give the vaccine, what does that mean? There is no structure.’ (Kenyan consultant, KC04)

In Uganda, rotations were previously 3 months in four major specialties but a policy in 2018 requires interns to rotate 5 months each in two specialties and 1 month each in two others (the 5–1 policy). While some interns appreciated this policy, most were unsatisfied as they were not able to fully acquire all the skills needed within 1 month in the minor specialty.

‘For instance I did 5 months of paed, one month of internal med…so recently I went for an interview and most of the questions they asked me were internal medicine questions where I spent only one month so I didn't pass the interview and personally I blame the 5–1.’ (Ugandan medical officer, UMO15)

## Discussion

Through analysing quantitative data collected from 845 junior MOs and qualitative data from 68 semistructured interviews with junior MOs and consultants, we provided an overview of the internship experience of junior medical doctors in Kenya and Uganda. Most of the quantitative and qualitative findings across different themes are consistent. Interns are generally satisfied with their job but many reported suffering from negative well-being exacerbated by working unreasonable hours due to staff shortages and being affected by the challenging working environment such as a lack of adequate resources and poor safety climate, the COVID-19 pandemic and health worker’s strikes. As for supervision, quantitative survey results suggested that most interns were satisfied with their supervision, however, in qualitative work many interviewees highlighted challenging scenarios where they had poor supervision and insufficient support due to consultants not being available. Such differences were also apparent in the recent Ugandan study.^34^ Bullying and racial discrimination from consultants were also sometimes reported in interviews. These findings highlighted the challenging internship experiences for medical doctors in Kenya and Uganda, which negatively impacted their learning, threatened individuals’ well-being and also the quality of care being delivered since interns are often at the front line of patient management. We also added to the global literature on internship experiences of medical doctors especially considering the scarcity of studies conducted in LMICs.

Over half of the junior MOs surveyed experienced negative well-being including signs of stress and burn-out. Internship training is stressful due to the quick transition from undergraduate supervised learning to being the frontline doctor, and many previous surveys also suggest that depression and burn-out are prevalent among the intern population: 26% interns experienced depression in a US cohort of 740 interns using the Patient Health Questionnaire-9 scale^14^; 42% of 101 Irish interns surveyed experienced 2 of the 3 Maslach Burnout Inventory symptoms (emotional exhaustion, depersonalisation and a sense of low personal accomplishment)^11^; 43% of 159 Myanmar house officers had one or more MBI burn-out symptoms.^10^ A more recent survey from Uganda suggested that most of the medical, nursing and dental interns had moderate to severe stress citing work, finances, an unclear future and the poor work environment as common reasons.^34^ Our current survey did not use these tools or did not use them in their original forms (some questions were dropped during the survey development phase) therefore, we cannot analyse our data against recommended cut-offs. However, responses to several single questions and quotes from qualitative interviews indicated that interns faced similar negative well-being challenges. However, these issues are sometimes overlooked by supervisors who perceive that today’s interns are not as resilient as the older generation, and on many occasions we found no formal support or counselling mechanism. Interestingly the government in Kenya did initiate a telecounselling and psychological care hotline during the pandemic,^44^ but it was not identified by any interviewees as a source of support. More emphasis should be given to improving interns’ well-being both from an individual’s and system’s perspectives.

Results on supervision and the teams interns work in gained from surveys and interviews were inconsistent. Survey data were more positive but interviews highlighted many challenging scenarios of lack of supervision or poor supervision. Such differences in quantitative and qualitative data could be due to important epistemological differences, response and central tendency bias of the survey data, a focus on ‘extreme cases’ in interview data, or other reasons. Such inconsistencies were also apparent in the recent Ugandan study.^34^ In another study of Kenyan interns, they reported that supervision was inadequate whereas supervisors felt that ‘they provided adequate supervision and that interns lacked the ability to initiate communication with them’.^35^ Nonetheless, these findings suggested that supervision and teamwork should be improved especially in the cases of hospitals with a general shortage of staff, and supervisors should also be trained on how to better lead and support interns.

Focus should be given to improving hospitals’ safety climate. Many doctors did not know the proper channels to raise concerns about personal and patient safety, and many are not confident there is a no-blame culture in their hospitals. Fear that nothing would change, not wanting to be a fuss and that reporting may have a negative effect on careers are common reasons for not speaking up about patient safety.^45 46^ This is perhaps especially for interns who need their supervisors to sign their logbooks, and where providing a ‘blame-free’ environment is still not officially legalised and widely acknowledged in policy,^47 48^ which should be improved in the long run.

As for physical resources at the internship hospitals, interns reported poor practice conditions especially the lack of adequate resources for patient care such as medication, diagnostics and equipment. This is consistent with other work that directly assessed the resource capacity of internship hospitals in Kenya.^49^ The lack of resources led to certain patients being referred to other hospitals thus interns not being able to acquire relevant skills, or interns forced to improvise threatening patient safety. The lack of suitable accommodation for interns also posed challenges to their personal safety as interns move between home and hospitals. Ministries of Health and regulators should ensure interns have no concerns about their personal safety.

It is noteworthy that Kenyan interns who worked in level 4 small hospitals had lower scores for well-being, supervision, working hours, patient safety and resource availability. Our data also indicated that interns in these smaller hospitals are more likely to be overworked, for as long as 72 hours on call, as the general staffing of the hospitals is low. This would likely lead to burn-out and other psychological problems. In comparison, Ugandan interns who worked in general hospitals perceived their educational and work environment better than their counterparts in regional and national referral hospitals. Survey data may help target interventions to specific hospitals to ensure that interns have good experiences across facilities. Interestingly, we did not notice any patterns of differences for most questions in the survey between different genders, including questions on gender and other discrimination (see online supplemental appendix 5).

Our work was conducted during the COVID-19 pandemic. Inevitably, this may have affected internship training experiences. Although data elsewhere suggested that inpatient admission in Kenya actually decreased during COVID-19,^50^ the studied cohort of interns may have experienced a higher level of stress and workload working in COVID-19 contexts while experiencing broader challenges such as health worker strikes. For others, COVID-19 lockdowns led to their facilities being closed and they were not able to fully gain the clinical exposure they expected.

Aside from improving resource availability and capacity of internship hospitals, our analyses highlight several other implications for Kenyan and Ugandan policy-makers. There is a need for a consultative planning and monitoring process by the government to review the medical internship process. More emphasis should be given to improving interns’ well-being. Regulators, educators and hospital administrators should address interns’ well-being through whole-system and individual actions. On a system level, educators and hospital administrators should be trained on how to better lead and support interns’ health and well-being, including the promotion of mental health awareness, developing skills in responding to interns’ health and well-being challenges, and more broadly improving learning and working environments, including preventing workplace bias and bullying.^51 52^ Establishing psychologist-facilitated support groups, counselling centres or peer support groups to help interns cope better might be useful.^53^ On an individual level, based on recommendations from MO and interns themselves, training institutions could do more to prepare graduating students for their internship perhaps through more formal induction periods and even offering resilience and coping strategy training. Leveraging and adapting experiences from other countries including high-income countries could be helpful for this area though it should be acknowledged that there is no one-size-fits-all solution and some of the interventions like Schwartz Rounds have not yet been piloted in LMICs.^51 54–56^ Regulators should also standardise and provide more training and support to supervisors and hospital administrators on how to better supervise and support interns. This might include internship coordinators and other hospital administrators reducing workplace hierarchy and empowering interns, improving interprofessional teamwork and promoting an active reporting culture not only to create a better learning environment but also to improve patient safety.^57^ These findings could also be relevant to other cadres undertaking internships such as pharmacists, dentists, nurses and clinical officers, and should be noticed by relevant policy-makers.

While this study provided a more complete description of the internship experience of junior doctors in Kenya and Uganda and contributed to the global literature on medical internship experiences, several limitations should be considered when interpreting these results. In terms of our sample recruitment, first we employed different recruitment strategies for the quantitative and qualitative components in each country, for example, the questionnaire survey used a convenience and snowball sampling approach, therefore, it is not fully representative of the intern population in respective countries and direct comparison between them is not feasible. Second, we included 196 current interns in our questionnaire survey and some of those interns are still in their first 6 months of internship (23%), and their assessment might change as they further rotate into other specialties. These interns mostly came from Kenya as the Uganda data collection started immediately after one cohort of interns just completed their internship. We also included junior MOs who finished their internships in 2018 and 2019 and had a 2–3 years recall period. Further analysis as shown in online supplemental appendix did not identify any significant pattern of difference between annual cohorts suggesting recall bias. Third, for the qualitative work, due to data collection logistics (as it was conducted within a shorter period) we had a smaller sample size in Uganda than in Kenya and some interviews collected in Uganda did not have as rich data, this might have some impact on data saturation and validity. Fourth, a few participants for qualitative and quantitative work are overlapping as those who participated in the survey work are eligible for interviews and vice versa, as we aimed to use survey to measure the ‘extent’ of internship experience challenges with larger sample size and use interviews to understand how and why more in-depth, however, this might have to biases and repetition. Lastly, for our data collection, as the questionnaire survey was part of scale development and validation work reported elsewhere^37^ we extensively revised question sets from existing tools. We did this based on a careful and collaborative process with our target population and stakeholders. Therefore, although the original cut-offs and rating systems could not be used and direct comparison with other studies is not achievable we feel our findings are useful. As our interest was focused on the internship experience we did not include questions on broader issues like the sociopolitical context that could have been important. Nonetheless, the quantitative survey, alongside qualitative data which is broader in focus, provided a rich description of the internship experiences and highlighted challenges with interns’ experiences.

## Conclusion

We highlight specific challenges for Kenyan and Ugandan interns. Intern surveys suggest most are satisfied and proud of their jobs but suffer from burn-out and stress due to working unreasonable hours and coping with the challenging working environment such as lack of adequate resources and poor safety climate. They also tended to receive inadequate support due to lack or poor quality of supervision, and sometimes supervisors were not available. These led to poor internship experiences which negatively impacted their learning, threatened individuals’ well-being and also the quality of care being delivered.

## Data Availability

Data are available on reasonable request. The datasets generated during and/or analysed during the current study are available from the corresponding author on reasonable request.
