# Supplementary material for: ‘We were treated like we are nobody’: a mixed-methods study of medical doctors’ internship experiences in Kenya and Uganda
Source: BMJ Glob Health. 2023 Nov 8;8(11):e013398. doi: 10.1136/bmjgh-2023-013398 (PMC10632815; doi:10.1136/bmjgh-2023-013398)
Supplement: Supplementary data [file bmjgh-2023-013398supp001.pdf]

Supplementary appendix 1. Survey instrument

Identification #

Please fill out this page together with the survey distributer.

#	Question	Enter or circle your answer
O1	What of the following best describe your current role?	1 = Medical student (I have not yet started my internship)<br>2 = Intern → ***Please answer O2***<br>3 = Medical doctor (I have finished my internship) → ***Please answer O2***
O2	How many months of internship training have you completed?	☐☐ (mm)
O3	When did you finish your internship training?	☐☐☐☐ (yyyy)

The questions in this section will ask you about your feelings and thoughts during your internship year(s). It also ask about your experiences, both positive and negative, as a helper: When you help people, you have direct contact with their lives. As you may have found, your compassion for those you help can affect you in positive and negative ways.
Please reflect on your whole internship experience in all department rotations rather than your current department rotation.
Please circle how often you felt or thought a certain way.

	**During your internship year, how often do you experience these feelings or thoughts?**	**5 = Very Often**	**4 = Often**	**3 = Sometimes**	**2 = Rarely**	**1 = Never**	**Don't know/ Prefer not to say**
Q1	I feel that I am unable to balance my work and personal life during my internship.	5	4	3	2	1	X
Q2	I feel nervous and/or stressed because of my internship work.	5	4	3	2	1	X
Q3	I feel confident about my ability to handle my personal problems during my internship.	5	4	3	2	1	X
Q4	I have felt that things have not gone my way in my life.	5	4	3	2	1	X
Q5	I find that I could not cope with all the work that I had to do during my internship.	5	4	3	2	1	X
Q6	I am able to control irritations in my work and life during my internship.	5	4	3	2	1	X
Q7	I feel that I am on top of things at work.	5	4	3	2	1	X
Q8	I am angered because of things that were outside of my control.	5	4	3	2	1	X
Q9	I feel that difficulties at work are piling up so high that I could not overcome them.	5	4	3	2	1	X
Q10	I have little interest or pleasure in doing things that I used to enjoy.	5	4	3	2	1	X
Q11	I feel down, depressed, or hopeless because of my internship work.	5	4	3	2	1	X
Q12	I have sleeping problems, either have trouble falling or staying asleep, or sleeping too much.	5	4	3	2	1	X
Q13	I feel tired or having little energy during my internship.	5	4	3	2	1	X
Q14	I have eating problems, either have poor appetite, or have been overeating.	5	4	3	2	1	X

	During your internship year, how often do you experience these feelings or thoughts?	5 = Very Often	4 = Often	3 = Sometimes	2 = Rarely	1 = Never	Don't know/ Prefer not to say
Q15	I have trouble concentrating on things either work-related, or outside of my work.	5	4	3	2	1	X
Q16	I have the thoughts that I would be better off dead or of hurting myself in some way.	5	4	3	2	1	X
Q17	I get satisfaction from being able to help people.	5	4	3	2	1	X
Q18	I feel invigorated and energized after work during my internship.	5	4	3	2	1	X
Q19	I like my work as a medical intern.	5	4	3	2	1	X
Q20	My ability to keep up with clinical techniques and protocols makes me feel pleased.	5	4	3	2	1	X
Q21	My internship work makes me feel satisfied.	5	4	3	2	1	X
Q22	I believe I can make a difference through my work.	5	4	3	2	1	X
Q23	I am proud of what I can do to help as a medical intern.	5	4	3	2	1	X
Q24	I believe that I am a success as a medical intern.	5	4	3	2	1	X
Q25	I am happy that I chose to do this work.	5	4	3	2	1	X
Q26	My internship experience met my expectation.	5	4	3	2	1	X
Q27	I am not as productive at work because I am losing sleep over work-related traumatic experiences.	5	4	3	2	1	X
Q28	I feel trapped by my job as a medical intern.	5	4	3	2	1	X
Q29	I feel worn out because of my work as a medical intern.	5	4	3	2	1	X
Q30	I feel overwhelmed because my case workload seems endless during the internship.	5	4	3	2	1	X
Q31	I feel bogged down and held back by the internship hospital.	5	4	3	2	1	X
Q32	I am preoccupied by concerns about multiple patients during my internship.	5	4	3	2	1	X
Q33	I find it difficult to separate my personal life from my life as a medical intern.	5	4	3	2	1	X

	During your internship year, how often do you experience these feelings or thoughts?	5 = Very Often	4 = Often	3 = Sometimes	2 = Rarely	1 = Never	Don't know/ Prefer not to say
Q34	I have been affected by the hardship and stress experienced by those patients I help.	5	4	3	2	1	X
Q35	I have felt on edge about various things because of my work helping patients.	5	4	3	2	1	X
Q36	I avoid certain activities or situations because they remind me of frightening experiences of the patients I help.	5	4	3	2	1	X
Q37	As a result of my helping, I have intrusive, frightening thoughts.	5	4	3	2	1	X

This section asks your perception of the internship training centre(s) you are currently affiliated with or previously affiliated with. It also asks your perception of the clinical supervisors, i.e. those who provided teaching and supervision for you during the internship period.
Please circle to what extent do you agree with these statements.

	To what extent do you agree with the following statements?	5 = Strongly agree	4 = Agree	3 = Neutral	2 = Disagree	1 = Strongly disagree	Don't know/ Prefer not to say
Q38	My clinical supervisors have set clear expectations	5	4	3	2	1	X
Q39	I have good clinical supervision at all times during my internship.	5	4	3	2	1	X
Q40	Time for my own reading, training and education is allocated by the hospital during my internship.	5	4	3	2	1	X
Q41	My clinical supervisors have good communication skills.	5	4	3	2	1	X
Q42	I am able to participate actively in educational sessions (e.g. continueing medical educations) during my internship.	5	4	3	2	1	X
Q43	My clinical supervisors are enthusiastic about teaching and supervision.	5	4	3	2	1	X
Q44	I have access to educational sessions and programmes that are relevant to my needs during my internship	5	4	3	2	1	X

	**To what extent do you agree with the following statements?**	**5 = Strongly agree**	**4 = Agree**	**3 = Neutral**	**2 = Disagree**	**1 = Strongly disagree**	**Don't know/ Prefer not to say**
Q45	The clinical supervisors provide me with regular feedback.	5	4	3	2	1	X
Q46	I have enough clinical learning opportunities for my needs during the internship period.	5	4	3	2	1	X
Q47	My clinical supervisors are accessible for teaching and supervision.	5	4	3	2	1	X
Q48	My clinical supervisors encourage me to be an independent learner.	5	4	3	2	1	X
Q49	The clinical supervisors provide me with feedback on my strengths and weaknesses to ensure my professional development.	5	4	3	2	1	X
Q50	My clinical supervisors have good mentoring skills.	5	4	3	2	1	X
Q51	I have opportunities to acquire the appropriate practical procedures for clinical practice during my internship.	5	4	3	2	1	X
Q52	My internship training makes me feel ready to be an independent medical practitioner.	5	4	3	2	1	X
Q53	My clinical supervisors promote an atmosphere of mutual respect.	5	4	3	2	1	X
Q54	I am pre-occupied with administrative work that impeded my ability to learn.	5	4	3	2	1	X
Q55	I had an informative internship induction programme.	5	4	3	2	1	X
Q56	There is an informative and comprehensive internship guideline, log book or clinical diary.	5	4	3	2	1	X
Q57	I have the appropriate level of responsibility as a medical intern.	5	4	3	2	1	X
Q58	I feel part of a team working here.	5	4	3	2	1	X

Q59	There are clear clinical protocols and guidelines across all departments in the internship hospital.	5	4	3	2	1	X
Q60	I have the opportunity to provide continuity of care for patients during my internship.	5	4	3	2	1	X
Q61	I have good collaboration with other medical practitioners, interns and clinical staff.	5	4	3	2	1	X
	**To what extent do you agree with the following statements?**	**5 = Strongly agree**	**4 = Agree**	**3 = Neutral**	**2 = Disagree**	**1 = Strongly disagree**	**Don't know/ Prefer not to say**
Q62	I have suitable access to careers advice services or advisors during my internship.	5	4	3	2	1	X
Q63	There are good counselling opportunities for medical interns who fail to complete their training satisfactorily.	5	4	3	2	1	X
Q64	I have a contract of employment or other document that provides information about hours of work during my internship.	5	4	3	2	1	X
Q65	My work hours are appropriate during my internship.	5	4	3	2	1	X
Q66	My workload is reasonable during my internship.	5	4	3	2	1	X
Q67	I am bleeped or called concerning the patients inappropriately during my internship.	5	4	3	2	1	X
Q68	I have to perform inappropriate tasks during my internship.	5	4	3	2	1	X
Q69	There is gender discrimination in my internship hospital.	5	4	3	2	1	X
Q70	There are other forms of discrimination (e.g. ethnicity, religion, tribe, disability) in my internship hospital.	5	4	3	2	1	X
Q71	There is a no-blame culture in my internship hospital.	5	4	3	2	1	X
Q72	I would feel safe being treated as a patient in my internship hospital.	5	4	3	2	1	X
Q73	Medical errors are handled appropriately in my internship hospital.	5	4	3	2	1	X

Q74	I know the proper channels to direct questions regarding patient safety.	5	4	3	2	1	X
Q75	It is difficult to discuss medical errors in my internship hospital.	5	4	3	2	1	X
Q76	I am encouraged by my colleagues to report any patient safety concerns I may have.	5	4	3	2	1	X
Q77	The culture in my internship hospital makes it easy to learn from the errors of others.	5	4	3	2	1	X
	**To what extent do you agree with the following statements?**	**5 = Strongly agree**	**4 = Agree**	**3 = Neutral**	**2 = Disagree**	**1 = Strongly disagree**	**Don't know/ Prefer not to say**
Q78	I know the proper channels to direct questions regarding my own safety.	5	4	3	2	1	X
Q79	I feel emotionally vulnerable within my internship hospital environment	5	4	3	2	1	X
Q80	I feel physically safe within my internship hospital.	5	4	3	2	1	X
Q81	I get bullied or victimized within my internship hospital.	5	4	3	2	1	X
Q82	There are clear and updated patient safety protocols in the internship hospital.	5	4	3	2	1	X
Q83	There are adequate infection prevention and control measures.	5	4	3	2	1	X
Q84	I can report any concern and receive responsive feedback in my internship hospital.	5	4	3	2	1	X
Q85	The internship hospital has good quality accommodation for me when on call.	5	4	3	2	1	X
Q86	There are adequate catering services provided by the internship hospital when I am on call.	5	4	3	2	1	X
Q87	The internship hospital has good internet connection for my study and work need.	5	4	3	2	1	X
Q88	The internship hospital has adequate supply of diagnostics, equipment and medication for my study and work need.	5	4	3	2	1	X

The questions in this section will ask you about your personal information. Please enter or circle you answer.

#	Question	Enter or circle your answer
D1	What is your gender?	1 = Male 2 = Female 3 = Others/prefer not to disclose
D2	What is your birth year?	□□□□ (yyyy)
D3	What is your marital status?<br><br>*(Circle one category)*	1=Single<br>2=Married<br>3=Divorced<br>4=Widowed<br>5=Separated
D4	Do you have children?	1=Yes 2=No

Supplementary appendix 2. Semi-structured interview guide

Questions (and probes) for medical officers:

1. Please tell us about yourself (refer to the spreadsheet)
    a. What's your current position? What type of occupation is it?
    b. Where did you complete your internship? Was it a public, private or mission hospital?
    c. Where did you undertake your undergraduate studies? Was it public or private?

2. How would you describe your internship experience?
    a. What are some of the things you have enjoyed most about your internship? Things least enjoyed? Can you give examples?
    b. Have you come across the term burnout, what does it mean to you? (state of emotional, physical and mental exhaustion caused by excessive and prolonged stress)
    c. How would you describe the level of support you received during internship? From whom?
    d. How do you think of patient safety in your internship hospital?
    e. How would you describe the level of preparation you got in medical school compared to the tasks you were given in your internship?
    f. How is it different from your intern friends who work in different hospitals?
    g. Did Covid impact your internship? health worker strike? government decentralization?
    h. What do you think can be done to improve your internship training and experience?

3. Can you tell me how did you choose to work in this current hospital/institution? What are the factors that influenced your decision to work here?
    a. Is this hospital/institution your first choice? Did you also apply to other jobs? Is it hard to find a job? How long did it take?
    b. Do you think there is a difference in terms of reputation of public/private/faith-based hospitals? How does that influence your decision?
    c. Did your preference for future career change during medical school and internship?

4. How did your internship experience influence your career decision?
    a. Do you prefer to work in certain hospitals or institutions because of your internship?
    b. How did your interaction with consultants/supervisors during the internship influenced your future plan?
    c. Did your previous relationship with your other colleagues during the internship hospital influenced your career decision?
    d. Are there any other social or political factors that influenced your career decision?

5. How safe do you feel about your current work environment, for you and for your patients?
    a. Your personal workload (any challenges accomplishing this?)
    b. In terms of the culture of safety in the workplace, and protocols if any (protective gear, waste disposal, essential work supplies and sundries, any changes with COVID-19)
    c. Adequacy of communication between colleagues for the care of patients (especially from senior to juniors, across professional boundaries e.g doctors to nurses)
    d. Teamwork (collaboration, cooperation, rather than competition)
    e. Participation in leadership decisions
    f. How and what would you like to see changed regarding safety at your workplace?

Questions (and probes) for consultants:

1. Please tell us about yourself
    a. What's your current position? What specialty are you in?
    b. What hospital are you in? Is it a public, private or mission hospital?

c. How long have you worked here?
d. How many interns have you supervised? How many are you supervising now?

2. What do you think of the quality of the incoming interns? How well do you feel medical schools prepare interns for the job they have undertaken as an intern?
    a. What could be done to better prep students for internship?
    b. From your experience have you noticed any variations/differences in how well interns are prepared for their internship (i.e. Public/private universities or locally trained/those who trained abroad?)
    c. What should med schools do to better prepare students for their internship?

3. Do you think the interns are competent as a general medical officer after this one-year internship training?
    a. As a consultant/supervisor do you ensure interns have accomplished this?
    b. Do they have adequate opportunities to practice during the internship?
    c. How do you usually supervise and teach the interns?
    d. Do you receive any support from the hospital as a supervisor?

4. What do you hear about the experience of medical interns more generally?
    a. Some interns report high level of burnout and inadequate support received. Do you think that's common? Can you give some example?
    b. How do you think the work conditions in this centre influence the learning experience of interns?
    c. What systems/structures are there to support interns during their internship? What types of support do you think should be provided to medical officer interns?
    d. Did Covid impact their internship? health worker strike? government decentralization?
    e. How was internship during your time? what is different now?
    f. What recommendations can you give to better prepare interns for their internship tasks?

5. How do you think interns decide on their future career after their internship? Why do you think interns choose to work in public/private/faith-based hospitals after they are registered and licensed?
    a. Why many interns opt for private practice?
    b. What do you think of the reputation of these hospitals?
    c. Is it hard for interns to find a job now? How long does it take to find a job?
    d. Are you involved in recruiting for this hospital? How does recruitment happen?

6. Can you broadly comment about safety at your current work environment, for you, your workers and for your patients?
    a. Your personal workload (any challenges accomplishing this?)
    b. In terms of the culture of safety in the workplace, and protocols if any (protective gear, waste disposal, essential work supplies and sundries, any changes with COVID-19)
    c. Adequacy of communication between colleagues for the care of patients (especially from senior to juniors, across professional boundaries e.g doctors to nurses)
    d. Teamwork (collaboration, cooperation, rather than competition)
    e. Participation in leadership decisions
    f. How and what would you like to see changed regarding safety at your workplace?

Supplementary appendix 3. Sample recruitment strategy

We used a mix of convenience and snowball sampling approach for survey and interview sampling. Data collection in Kenya started in June 2021 and ended in Sept 2021 for interview and started in Nov 2021 and ended in May 2022 for survey; in Uganda started in April 2022 and ended in June 2022 for both interview and survey.

In Kenya, for survey administration, we engaged with a number of different stakeholders such as the Kenyan Medical Practitioners and Dentists Council, Kenya Medical Association Young Doctor Network, Kenya Young Doctor Caucus, three major medical schools and selected facilities in the Clinical Information Network operated by KEMRI-Wellcome Trust Research Programme, and asked them to share the survey through their respective platforms such as WhatsApp or SMS. The survey is anonymous aside from participants could provide their phone number and email addresses if they would like to be reimbursed of their data use. For semi-structured interviews, we identified several MOs and consultants through our study group network, and then asked them to further introduce us to more eligible participants in Kenya. The interviewees remain anonymous as we only collected their basic characteristics below.

In Uganda, for survey the Federation for Uganda Medical Interns and the Uganda Medical Association were used for circulation of recruitment materials. The research team also physically visited five health facilities based in Kampala, and two major internship training sites upcountry and asked MO interns and junior MOs who work in these facilities to fill out the survey. At the time of data collection, one cohort of interns had just completed internship. For semi-structured interviews, recruitment was conducted by research assistants through health facility visits. Both interview and survey were anonymous in Uganda.

Supplementary appendix 4. Semi-structured interview sample characteristics

**Junior medical officers**	**Kenya (n=30)**	**Uganda (n=24)**
Internship hospital type	25 public, 3 private not for profit, 1 private, 1 military	14 public, 8 private not for profit, 2 private
Internship hospital level	14 level 5, 16 level 4	3 national referral, 11 regional referral, 10 general
Current occupation	26 Mo, 2 researcher, 1 resident, 1 in business	14 MO, 2 researcher, 1 resident, 7 current unemployed
**Consultants**	**Kenya (n=10)**	**Uganda (n=4)**
Internship hospital type	8 public, 1 private not for profit, 1 private	3 public, 1 private not for profit
Internship hospital level	3 level 6, 4 level 5, 3 level 4	3 national, 1 general
Specialty	2 surgery, 2 int med, 5 paediatric, 1 OBGYN	2 OBGYN, 1 surgery, 1 paediatric

Supplementary appendix 5. Full survey results by hospital administrative level, gender and years of graduation

		Kenya										Uganda									
		**Level 4 small hospitals (n=99)**	**Level 4 large hospitals (n=98)**	**Level 5&6 hospitals (n=137)**	**Other hospitals (n=24)**	**Male (n=183)**	**Female (n=170)**	**Current intern (n=131)**	**Finished between 2020 and 2021 (n=166)**	**Finished between 2018 and 2019 (n=61)**	**Total (n=358)**	**General (n=117)**	**Regional referral (n=230)**	**National referral (n=122)**	**Other hospitals (n=18)**	**Male (n=336)**	**Female (n=150)**	**Current intern (n=65)**	**Finished between 2020 and 2021 (n=348)**	**Finished between 2018 and 2019 (n=74)**	**Total (n=487**
q1	(-) I feel that I am unable to balance my work and personal life during my internship.	59%	58%	64%	79%	60%	64%	56%	67%	62%	62%	47%	48%	53%	50%	48%	51%	51%	49%	46%	49%
q2	(-) I feel nervous and/or stressed because of my internship work.	66%	49%	61%	58%	55%	63%	53%	62%	64%	59%	44%	35%	40%	33%	38%	39%	31%	40%	38%	38%
q3	I feel confident about my ability to handle my personal problems during my internship.	41%	50%	40%	38%	50%	36%	47%	39%	48%	43%	54%	60%	54%	67%	57%	58%	48%	61%	49%	57%
q4	(-) I have felt that things have not gone my way in my life.	31%	24%	33%	46%	32%	29%	26%	34%	31%	31%	21%	14%	23%	6%	17%	19%	20%	18%	15%	18%
q5	(-) I find that I could not cope with all the work that I had to do during my internship.	39%	33%	36%	42%	41%	32%	37%	33%	44%	36%	25%	19%	26%	28%	24%	20%	22%	22%	23%	22%
q6	I am able to control irritations in my work and life during my internship.	56%	56%	52%	38%	56%	51%	50%	51%	64%	53%	59%	64%	65%	50%	63%	61%	65%	65%	47%	62%
q7	I feel that I am on top of things at work.	44%	44%	48%	42%	47%	45%	47%	45%	46%	46%	50%	51%	38%	61%	45%	54%	32%	51%	47%	48%
q8	(-) I am angered because of things that were outside of my control.	57%	54%	45%	38%	54%	45%	47%	51%	53%	50%	27%	27%	26%	28%	26%	28%	26%	26%	27%	27%
q9	(-) I feel that difficulties at work are piling up so high that I could not overcome them.	30%	25%	30%	25%	32%	25%	28%	28%	31%	28%	18%	14%	15%	17%	16%	11%	12%	15%	16%	15%
q10	(-) I have little interest or pleasure in doing things that I used to enjoy.	23%	25%	27%	46%	22%	30%	29%	27%	21%	27%	28%	19%	19%	28%	22%	21%	28%	22%	16%	22%
q11	(-) I feel down, depressed, or hopeless because of my internship work.	26%	18%	27%	29%	21%	28%	25%	25%	23%	25%	18%	10%	12%	11%	13%	11%	17%	12%	12%	12%
q12	(-) I have sleeping problems, either have trouble falling or staying asleep, or sleeping too much.	37%	36%	35%	50%	31%	42%	34%	39%	38%	37%	25%	18%	27%	11%	22%	21%	25%	21%	20%	22%
q13	(-) I feel tired or having little energy during my internship.	52%	48%	54%	63%	47%	57%	48%	55%	54%	52%	38%	22%	25%	39%	26%	29%	29%	28%	22%	27%
q14	(-) I have eating problems, either have poor appetite, or have been overeating.	42%	34%	47%	38%	34%	48%	35%	45%	48%	42%	27%	13%	22%	22%	17%	23%	23%	20%	12%	19%
q15	(-) I have trouble concentrating on things either work-related, or outside of my work.	29%	18%	26%	17%	23%	25%	25%	23%	25%	24%	13%	9%	13%	0%	10%	11%	19%	9%	12%	11%
q16	(-) I have the thoughts that I would be better off dead or	11%	5%	7%	4%	8%	8%	9%	7%	7%	8%	9%	2%	5%	6%	4%	5%	11%	4%	3%	5%

	of hurting myself in some way.																				
q17	I get satisfaction from being able to help people.	80%	87%	88%	88%	89%	82%	81%	89%	87%	86%	80%	87%	86%	94%	88%	80%	72%	89%	80%	85%
q18	I feel invigorated and energized after work during my internship.	17%	33%	26%	29%	31%	21%	26%	25%	28%	26%	47%	41%	43%	50%	47%	34%	46%	43%	41%	43%
q19	I like my work as a medical intern.	44%	58%	47%	54%	53%	47%	57%	46%	46%	50%	62%	71%	64%	72%	69%	65%	71%	68%	62%	67%
q20	My ability to keep up with clinical techniques and protocols makes me feel pleased.	70%	80%	75%	71%	75%	73%	73%	76%	74%	74%	80%	87%	83%	94%	86%	82%	77%	85%	87%	84%
q21	My internship work makes me feel satisfied.	54%	66%	59%	50%	63%	55%	59%	60%	57%	59%	67%	75%	66%	89%	72%	69%	69%	73%	66%	71%
q22	I believe I can make a difference through my work.	75%	86%	74%	63%	79%	74%	74%	79%	75%	77%	84%	87%	86%	100%	87%	85%	79%	89%	82%	86%
q23	I am proud of what I can do to help as a medical intern.	81%	84%	81%	83%	85%	78%	79%	86%	79%	82%	89%	87%	83%	94%	85%	90%	75%	89%	85%	87%
q24	I believe that I am a success as a medical intern.	73%	71%	80%	63%	78%	72%	70%	80%	72%	75%	77%	84%	79%	83%	83%	77%	68%	84%	78%	81%
q25	I am happy that I chose to do this work.	55%	68%	64%	58%	69%	56%	60%	63%	64%	62%	76%	77%	79%	78%	78%	76%	83%	78%	70%	77%
q26	My internship experience met my expectation.	38%	54%	47%	42%	53%	41%	45%	46%	49%	46%	63%	61%	57%	56%	62%	58%	52%	62%	58%	60%
q27	(-) I am not as productive at work because I am losing sleep over work-related traumatic experiences.	24%	18%	21%	21%	19%	24%	21%	21%	21%	21%	18%	9%	15%	0%	11%	14%	25%	10%	11%	12%
q28	(-) I feel trapped by my job as a medical intern.	34%	36%	32%	33%	34%	32%	40%	31%	28%	34%	28%	23%	31%	22%	28%	21%	32%	26%	23%	26%
q29	(-) I feel worn out because of my work as a medical intern.	58%	53%	56%	50%	53%	57%	50%	56%	64%	55%	45%	33%	44%	33%	40%	35%	35%	40%	37%	39%
q30	(-) I feel overwhelmed because my case workload seems endless during the internship.	56%	50%	61%	46%	54%	57%	45%	59%	69%	56%	44%	37%	46%	50%	42%	39%	40%	41%	43%	42%
q31	(-) I feel bogged down and held back by the internship hospital.	43%	28%	39%	38%	39%	34%	34%	39%	38%	37%	27%	18%	30%	28%	26%	17%	23%	23%	24%	23%
q32	(-) I am preoccupied by concerns about multiple patients during my internship.	59%	47%	58%	42%	56%	52%	47%	57%	64%	54%	39%	37%	39%	28%	36%	41%	45%	37%	35%	38%
q33	(-) I find it difficult to separate my personal life from my life as a medical intern.	44%	38%	48%	50%	46%	43%	39%	45%	54%	44%	35%	30%	38%	33%	36%	27%	48%	30%	34%	33%
q34	(-) I have been affected by the hardship and stress experienced by those patients I help.	35%	22%	36%	17%	32%	29%	29%	32%	31%	31%	23%	19%	25%	28%	21%	21%	26%	20%	24%	22%
q35	(-) I have felt on edge about various things because of my work helping patients.	40%	25%	35%	17%	33%	32%	30%	36%	28%	32%	22%	16%	30%	22%	25%	13%	22%	22%	19%	21%
q36	(-) I avoid certain activities or situations because they remind me of frightening experiences of the patients I help.	19%	18%	18%	13%	16%	19%	20%	18%	13%	18%	20%	9%	7%	22%	11%	14%	19%	10%	12%	12%

q37	(-) As a result of my helping, I have intrusive, frightening thoughts.	18%	14%	9%	0%	11%	14%	13%	10%	16%	12%	9%	6%	5%	6%	7%	5%	14%	4%	12%	6%
q38	My clinical supervisors have set clear expectations	61%	71%	68%	50%	64%	69%	61%	66%	74%	66%	88%	80%	79%	83%	81%	83%	88%	79%	88%	82%
q39	I have good clinical supervision at all times during my internship.	44%	61%	63%	58%	60%	55%	56%	56%	62%	57%	78%	61%	62%	78%	63%	72%	72%	66%	62%	66%
q40	Time for my own reading, training and education is allocated by the hospital during my internship.	13%	26%	18%	33%	18%	22%	21%	19%	18%	20%	37%	36%	19%	33%	30%	37%	32%	31%	34%	32%
q41	My clinical supervisors have good communication skills.	59%	64%	50%	50%	58%	55%	60%	55%	53%	56%	74%	74%	61%	94%	70%	76%	72%	70%	78%	72%
q42	I am able to participate actively in educational sessions (e.g. continueing medical educations) during my internship.	61%	67%	66%	67%	62%	67%	57%	69%	71%	65%	85%	88%	71%	78%	84%	81%	74%	85%	81%	83%
q43	My clinical supervisors are enthusiastic about teaching and supervision.	50%	63%	59%	63%	59%	58%	59%	59%	53%	58%	74%	65%	69%	89%	70%	68%	68%	71%	64%	69%
q44	I have access to educational sessions and programmes that are relevant to my needs during my internship	34%	43%	51%	46%	44%	44%	41%	45%	49%	44%	78%	68%	66%	78%	70%	71%	66%	73%	61%	70%
q45	The clinical supervisors provide me with regular feedback.	38%	49%	50%	58%	47%	48%	41%	52%	46%	47%	67%	61%	50%	50%	58%	61%	59%	57%	69%	59%
q46	I have enough clinical learning opportunities for my needs during the internship period.	54%	64%	66%	54%	61%	64%	60%	62%	64%	62%	81%	77%	66%	72%	75%	77%	69%	75%	81%	75%
q47	My clinical supervisors are accessible for teaching and supervision.	58%	65%	66%	63%	65%	63%	66%	61%	64%	63%	78%	70%	65%	83%	70%	74%	74%	71%	68%	71%
q48	My clinical supervisors encourage me to be an independent learner.	68%	76%	67%	71%	72%	69%	67%	70%	75%	70%	83%	78%	72%	83%	78%	77%	74%	80%	70%	78%
q49	The clinical supervisors provide me with feedback on my strengths and weaknesses to ensure my professional development.	46%	56%	56%	54%	57%	50%	53%	51%	56%	53%	74%	71%	56%	61%	67%	67%	74%	65%	73%	67%
q50	My clinical supervisors have good mentoring skills.	49%	56%	58%	58%	58%	54%	59%	53%	53%	55%	76%	71%	70%	78%	73%	70%	79%	71%	72%	72%
q51	I have opportunities to acquire the appropriate practical procedures for clinical practice during my internship.	73%	83%	73%	75%	80%	72%	75%	77%	75%	76%	86%	83%	73%	83%	82%	81%	85%	83%	72%	81%
q52	My internship training makes me feel ready to be an independent medical practitioner.	72%	82%	80%	71%	84%	72%	78%	78%	74%	77%	88%	89%	84%	89%	90%	82%	83%	89%	84%	88%
q53	My clinical supervisors promote an atmosphere of mutual respect.	59%	69%	56%	50%	62%	58%	63%	61%	53%	60%	79%	75%	71%	78%	75%	75%	80%	74%	74%	75%
q54	(-) I am pre-occupied with administrative work that impeded my ability to learn.	16%	16%	21%	17%	21%	14%	21%	16%	16%	18%	27%	14%	14%	11%	17%	17%	23%	14%	24%	17%

q55	I had an informative internship induction programme.	40%	43%	59%	50%	53%	45%	46%	51%	49%	49%	68%	52%	56%	56%	60%	49%	68%	55%	57%	57%
q56	There is an informative and comprehensive internship guideline, log book or clinical diary.	77%	86%	84%	83%	84%	81%	81%	83%	84%	82%	51%	41%	47%	33%	42%	50%	55%	42%	47%	45%
q57	I have the appropriate level of responsibility as a medical intern.	63%	67%	66%	71%	71%	60%	70%	65%	61%	66%	86%	82%	77%	94%	82%	83%	80%	84%	76%	82%
q58	I feel part of a team working here.	64%	75%	75%	67%	75%	67%	66%	74%	75%	71%	86%	91%	81%	94%	88%	87%	86%	89%	81%	88%
q59	There are clear clinical protocols and guidelines across all departments in the internship hospital.	33%	43%	57%	46%	48%	45%	42%	47%	51%	46%	77%	64%	66%	61%	68%	67%	77%	68%	58%	68%
q60	I have the opportunity to provide continuity of care for patients during my internship.	72%	81%	81%	79%	77%	81%	73%	83%	77%	78%	91%	86%	83%	89%	87%	84%	91%	87%	80%	86%
q61	I have good collaboration with other medical practitioners, interns and clinical staff.	85%	83%	83%	88%	84%	84%	80%	86%	85%	84%	93%	87%	84%	100%	89%	87%	89%	89%	82%	88%
q62	I have suitable access to careers advice services or advisors during my internship.	38%	34%	37%	50%	43%	32%	41%	36%	34%	37%	70%	61%	66%	78%	65%	67%	71%	65%	61%	65%
q63	There are good counselling opportunities for medical interns who fail to complete their training satisfactorily.	13%	24%	21%	38%	23%	19%	25%	16%	23%	21%	48%	43%	30%	33%	38%	46%	49%	38%	45%	40%
q64	I have a contract of employment or other document that provides information about hours of work during my internship.	25%	20%	29%	38%	28%	24%	26%	27%	25%	26%	50%	45%	46%	50%	46%	49%	52%	46%	45%	47%
q65	My work hours are appropriate during my internship.	15%	19%	20%	33%	22%	17%	23%	17%	18%	19%	41%	33%	31%	44%	35%	36%	43%	32%	41%	35%
q66	My workload is reasonable during my internship.	13%	28%	18%	50%	22%	22%	28%	18%	16%	22%	40%	30%	28%	39%	34%	29%	49%	28%	39%	32%
q67	(-) I am bleeped or called concerning the patients inappropriately during my internship.	54%	52%	47%	63%	56%	47%	44%	56%	53%	51%	45%	43%	38%	50%	47%	32%	46%	41%	45%	43%
q68	(-) I have to perform inappropriate tasks during my internship.	42%	32%	39%	54%	44%	35%	37%	41%	38%	39%	27%	32%	25%	33%	33%	21%	28%	29%	32%	29%
q69	(-) There is gender discrimination in my internship hospital.	17%	17%	14%	13%	13%	19%	15%	16%	16%	16%	10%	9%	15%	11%	13%	7%	22%	9%	10%	11%
q70	(-) There are other forms of discrimination (e.g. ethnicity, religion, tribe, disability) in my internship hospital.	26%	26%	27%	33%	26%	28%	24%	30%	23%	27%	21%	11%	17%	11%	17%	12%	25%	13%	18%	15%
q71	There is a no-blame culture in my internship hospital.	18%	21%	29%	17%	23%	24%	24%	21%	26%	23%	29%	28%	35%	39%	33%	26%	49%	27%	30%	31%
q72	I would feel safe being treated as a patient in my internship hospital.	29%	31%	49%	50%	39%	38%	36%	38%	46%	39%	73%	40%	41%	61%	52%	41%	51%	47%	54%	49%

q73	Medical errors are handled appropriately in my internship hospital.	43%	52%	53%	58%	51%	51%	50%	50%	54%	51%	72%	67%	50%	78%	66%	62%	66%	63%	68%	64%
q74	I know the proper channels to direct questions regarding patient safety.	55%	51%	61%	67%	61%	52%	59%	54%	59%	57%	77%	72%	65%	67%	72%	69%	72%	73%	64%	71%
q75	(-) It is difficult to discuss medical errors in my internship hospital.	41%	29%	33%	33%	34%	34%	36%	34%	30%	34%	20%	27%	30%	22%	29%	19%	23%	26%	26%	26%
q76	I am encouraged by my colleagues to report any patient safety concerns I may have.	46%	42%	56%	58%	54%	45%	47%	51%	51%	49%	69%	64%	63%	61%	66%	63%	72%	66%	54%	65%
q77	The culture in my internship hospital makes it easy to learn from the errors of others.	47%	55%	53%	50%	57%	47%	53%	51%	51%	52%	73%	75%	65%	83%	74%	69%	79%	71%	73%	72%
q78	I know the proper channels to direct questions regarding my own safety.	42%	43%	46%	58%	55%	34%	49%	44%	39%	45%	79%	68%	69%	89%	73%	68%	77%	72%	65%	72%
q79	(-) I feel emotionally vulnerable within my internship hospital environment	37%	37%	40%	33%	34%	41%	39%	38%	36%	38%	32%	25%	29%	17%	28%	25%	34%	29%	14%	27%
q80	I feel physically safe within my internship hospital.	67%	78%	72%	63%	75%	68%	72%	72%	69%	72%	80%	76%	67%	89%	75%	75%	74%	76%	73%	75%
q81	(-) I get bullied or victimized within my internship hospital.	29%	22%	27%	29%	23%	30%	23%	31%	23%	27%	15%	10%	16%	6%	15%	7%	20%	12%	11%	13%
q82	There are clear and updated patient safety protocols in the internship hospital.	26%	29%	47%	58%	42%	32%	39%	37%	31%	37%	68%	54%	56%	72%	58%	60%	62%	59%	55%	59%
q83	There are adequate infection prevention and control measures.	46%	54%	61%	71%	57%	54%	53%	55%	59%	55%	74%	63%	53%	78%	63%	63%	59%	64%	62%	63%
q84	I can report any concern and receive responsive feedback in my internship hospital.	35%	39%	41%	46%	43%	36%	47%	34%	34%	39%	74%	63%	57%	78%	64%	65%	83%	62%	62%	65%
q85	The internship hospital has good quality accommodation for me when on call.	23%	38%	36%	63%	34%	35%	35%	35%	33%	35%	73%	44%	33%	56%	44%	59%	48%	48%	49%	48%
q86	There are adequate catering services provided by the internship hospital when I am on call.	7%	18%	20%	38%	16%	19%	18%	18%	15%	17%	66%	27%	21%	11%	30%	43%	40%	33%	34%	34%
q87	The internship hospital has good internet connection for my study and work need.	16%	17%	27%	46%	25%	20%	24%	22%	21%	23%	51%	42%	16%	28%	32%	50%	39%	38%	32%	37%
q88	The internship hospital has adequate supply of diagnostics, equipment and medication for my study and work need.	21%	30%	42%	54%	34%	32%	34%	34%	33%	34%	70%	34%	32%	56%	40%	49%	46%	43%	39%	43%

Supplementary appendix 6. Structured reflexivity statement

Domain	Question	Answer
Study conceptualisation	1. How does this study address local research and policy priorities?	This research is a collaboration between Kenyan, Ugandan and international researchers. The research question is aligned with Kenyan and Uganda's human resources for health strategies and carefully discussed with Kenyan national Ministry of Health stakeholders.
	2. How were local researchers involved in study design?	The first authors are a UK-based Chinese researcher (YZ) and a Kenyan researcher (DM) who are not doctors but with research experiences in public health, and the senior authors are a Ugandan doctor and researcher (RT) and a UK pediatrician with extensive experiences working in Kenya (ME). These four co-designed the study including instrument development and data interpretation to promote rigour.
Research management	3. How has funding been used to support the local research team(s)?	This project leveraged a wide range of funding from UK and elsewhere to support the research team including funding for the Kenyan and Uganda core researchers to develop skills and networks with an aim to develop further work in this area.
Data acquisition and analysis	4. How are research staff who conducted data collection acknowledged?	Key research staff involved in coordinating and collecting data (both survey and interviews) have been listed as group authors.
	5. How have members of the research partnership been provided with access to study data?	The first authors and senior authors have access to all data, all other authors have access to aggregate-level data due to consideration for anonymity.
	6. How were data used to develop analytical skills within the partnership?	The first authors who are early career researchers have been supported by senior authors and the research group to develop analytical skills.
Data interpretation	7. How have research partners collaborated in interpreting study data?	All group authors were involved in interpreting the study data, this included several feedback meetings in both countries and a feedback workshop held in Kenya.
Drafting and revising for intellectual content	8. How were research partners supported to develop writing skills?	The first authors who are early career researchers have been supported by senior authors and the research group to develop writing skills.
